# Antiviral Inhibition of Enveloped Virus Release by Tetherin/BST-2: Action and Counteraction

**DOI:** 10.3390/v3050520

**Published:** 2011-05-06

**Authors:** Anna Le Tortorec, Suzanne Willey, Stuart J. D. Neil

**Affiliations:** Department of Infectious Disease, King’s College London School of Medicine, Guy’s Hospital, London SE1 9RT, UK; E-Mails: anna.le_tortorec@kcl.ac.uk (A.L.T.); suzanne.willey@kcl.ac.uk (S.W.)

**Keywords:** Tetherin/BST2, restriction of enveloped virus release, interferon, viral countermeasure, Vpu

## Abstract

Tetherin (BST2/CD317) has been recently recognized as a potent interferon-induced antiviral molecule that inhibits the release of diverse mammalian enveloped virus particles from infected cells. By targeting an immutable structure common to all these viruses, the virion membrane, evasion of this antiviral mechanism has necessitated the development of specific countermeasures that directly inhibit tetherin activity. Here we review our current understanding of the molecular basis of tetherin’s mode of action, the viral countermeasures that antagonize it, and how virus/tetherin interactions may affect viral transmission and pathogenicity.

## 1. Introduction

The induction of the interferon response by viral infection leads to a cellular “antiviral state”, in which various signaling pathways trigger the upregulation of an array of cellular genes involved in host defense. Some of these genes encode proteins that directly inhibit various stages of mammalian virus replication. In the last decade, several examples of host-encoded antiviral proteins that potently inhibit aspects of retroviral replication (so-called retroviral restriction factors) have been identified on the basis of the cell-type or species specificity of human immunodeficiency virus type 1 (HIV-1) replication. Of these proteins, members of the APOBEC3 family of cytidine deaminases and tetherin/BST-2 are particularly notable because they target molecular features of the virus that cannot be varied (the chemical nature of the genome or the lipid envelope), and have thus necessitated the development of virus-encoded countermeasures to evade them—the accessory genes Vif and Vpu, respectively [1].

Tetherin (bone marrow stromal cell antigen 2—BST-2, CD317) was discovered as the factor responsible for the defect in virion release of HIV-1 mutants lacking the accessory gene *vpu* [2,3]. In the absence of Vpu expression, cell-free HIV-1 particles are poorly released from CD4+ T cells and macrophages, and mature virions accumulate on the cell surface and in vacuolar structures [4,5]. It has subsequently become clear that tetherin’s unique mode of action allows it to target a wide range of mammalian enveloped viruses, and there are now several examples of viral proteins, which, like Vpu, specifically counteract this antiviral factor. In this review we will focus on the recent progress and future directions in our understanding of tetherin’s mechanism of action, how virally encoded countermeasures target its activity, and the potential role of these interactions in *in vivo* viral transmission and pathogenesis. While most of the studies so far focus on primate lentiviruses, we will draw attention to general principles likely to be applicable to many other enveloped viruses.

## 2. Tetherin/BST-2/CD317

Tetherin is widely expressed in response to type I IFN, and is also constitutively expressed on several cell types, including mature B cells, plasma cells and plasmacytoid dendritic cells [6]. It can also be upregulated on myeloid cells and lymphocytes by various activatory stimuli such as pro-inflammatory cytokines, and in ruminants is highly expressed in the endometrial stroma surrounding the conceptus [7]. Prior to the discovery of its role as an antiviral effector molecule, it had been designated as the tumor antigen HM1.24 due to its expression on multiple myeloma cells, and has been of interest in this regard as a target for cancer immunotherapy [8,9]. Its expression in the bone marrow stroma and on B cells links it to a suspected role in B cell development [8,9], and a recent report suggests a role in monocyte adhesion [10]. Besides its inhibition of virus particle release, the only other defined physiological function of tetherin is as a ligand for the leukocyte inhibitory receptor ILT7 in the modulation of Toll-like receptor function [11].

Tetherin orthologues have been identified in the genomes of all mammals analyzed to date, and of those tested all possess the ability to inhibit retroviral particle release [12–14]. Curiously, the tetherin gene was duplicated in ruminants prior to the diversion of sheep, goats and cows [7]. Both sheep orthologues have antiviral activity, although some differences exist in their relative potency [7]. Sequence analyses have demonstrated that tetherin, like many immunological effector molecules, has been under high levels of positive selection during mammalian evolution, particularly in areas of the protein implicated as targets for virally encoded countermeasures [12,15,16] (see below). These analyses, while differing in their interpretation of the relative levels of positive selection between domains of the tetherin protein, all suggest that tetherin evolution has been shaped by the constant interaction with viruses and their encoded antagonists.

### 

#### Tetherin Structure, Topology and Localization

**Figure 1:**
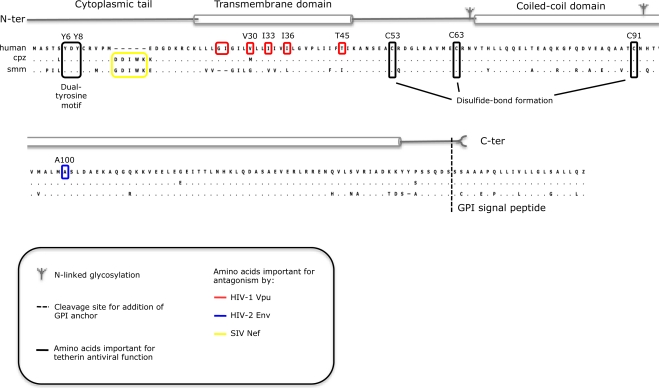
Features of tetherin. A schematic representation of the structural domains of tetherin is shown above an alignment of the human, chimpanzee (cpz) and sooty mangabey (smm) amino acid sequences. Black boxes around amino acids indicate regions important for the antiviral function of all three tetherin proteins. Red, blue and yellow boxes indicate amino acids important for the recognition and/or antagonism of tetherin by HIV-1 Vpu, HIV-2 Env and SIV Nef, respectively.

Tetherin is a small type II membrane protein of 181 amino acids with a molecular weight of between 29 and 33 kDa depending on its glycosylation state. It has an unusual topology with both ends embedded in the cellular membrane by two different types of membrane anchor: a transmembrane domain proximal to the N-terminus and a C-terminal glycosyl-phosphatidylinositol (GPI) anchor [17] (Figure 1). As yet the only other protein to show a similar topology is a minor isoform of the prion protein PrP [18].

The two membrane anchors are connected by the extracellular domain of tetherin, comprising an extended coiled-coil structure; the intracellular N terminus consists of a short cytoplasmic tail. The extracellular domain of tetherin contains two N-linked glycosylation sites, and mediates homodimerization through disulfide linkages formed by at least one of three cysteine residues [19,20]. Glycosylation contributes to the correct transport and folding of the protein [19]. Recently, partial X-ray crystallography structures of the extracellular domain of tetherin [21–24], have confirmed the presence of a parallel disulfide-linked dimeric, α-helical coiled-coil. The coiled-coil contains structural irregularities along its length that are predicted to confer considerable flexibility [21]. In the structure of the oxidized form of the human tetherin ectodomain, this N-terminal region is unresolved in the crystal, further suggesting conformational flexibility in this area [22].

Tetherin is located both at the plasma membrane and in intracellular compartments. At the plasma membrane, the GPI anchor embeds the C terminus of tetherin in cholesterol-rich microdomains, from which HIV-1 and other enveloped viruses preferentially bud [17,25–27]. The intracellular pool of tetherin is located predominantly in the trans-Golgi network (TGN), but is also found in early endosomes and potentially in recycling endosomes [6,17,26,28]. Trafficking of tetherin between the plasma membrane and the TGN requires a tyrosine-based sorting signal in the tetherin cytoplasmic tail. This YxYxxϕ motif recruits clathrin adaptor complexes and is highly conserved in all known tetherin proteins [26,29]. This site has been shown to interact with both clathrin adaptors AP1 and AP2. The AP2 complex delivers tetherin to early endosomes, and the AP1 complex then mediates transport of tetherin between these endosomes and the TGN [26,29].

In polarized epithelial cells tetherin localizes specifically to the apical surface. Knockdown of tetherin expression leads to disappearance of the underlying cortical actin network. This linkage between tetherin-containing microdomains and the actin cytoskeleton is mediated by BAR-RacGAP protein RICH2 [25]. Disruption of tetherin/RICH2 interactions leads to activation of Rac1, explaining the change in actin dynamics. However, what the physiological role of tetherin is in polarized cells remains to be determined.

## 3. Antiviral Activity of Tetherin

To date, tetherin has been shown to restrict the release of retroviruses (all classes) [7,30–32], filoviruses (Ebola virus and Marburg virus) [30,33,34], arenaviruses (Lassa virus and Machupo virus) [34,35], a paramyxovirus (Nipah virus) [35], gamma-herpesviruses (Kaposi’s sarcoma-associated herpesvirus (KSHV)) [36,37] and rhabdoviruses (vesicular stomatitis virus [38]). The ability of tetherin to target such a diverse group of viruses resides in its ability to target a feature common to them all: their host cell-derived lipid bilayer. This list of susceptible viruses is bound to grow, as in principal tetherin could target any enveloped virus that buds from cellular membrane enriched in tetherin.

Biochemical and structural evidence, mostly from studies of HIV-1, currently favours a direct tethering mechanism of Virus Res.triction in which parallel tetherin dimers physically crosslink virion and cellular/other virion membranes. This results in the characteristic retention of mature virions in protease sensitive layers on the plasma membrane [2,19].

EM studies reveal electron-dense tethers visible between the cell and the virus, or between two virions [2,39,40]. Furthermore, recent immune-electron microscopy studies demonstrate that tetherin is present between the cells and the virions, and is therefore likely to constitute the physical linkage responsible for attachment of nascent HIV-1 virions to the plasma membrane [19,28,40,41]. However, electron tomography studies will be required to provide conclusive evidence for a direct tethering mechanism. Partial protease stripping experiments demonstrated that tetherin is incorporated into HIV-1 virions in a parallel configuration, although additional conformations cannot be ruled out[19,40]. Interestingly, tetherin was found enriched on filamentous structures, far longer than the 17nm estimate of a single tetherin molecule, connecting virions to the plasma membrane [40]. The nature of these structures and their role in tethering virus particles at the plasma membrane remain to be clarified.

Studies using an artificial “tetherin” have demonstrated that it is the unique topology of tetherin, rather than the amino acid sequence, that confers its virus-tethering ability [19]. Remarkably, a molecule constructed from the dimeric N-terminus of the transferrin receptor, a coiled-coil motif from DMPK, and a GPI anchor from uPAR was able to obstruct virus release in the same manner as the native molecule [19]. This also suggests that that it is unlikely that a cellular co-factor is required for physical particle tethering.

Both dimerization of tetherin molecules and its GPI anchor are essential for restriction of retroviral particle release [2,19,20]. Moreover, interactions within the coiled-coil domain and at least one disulfide bond formation are required for dimer stability and HIV-1 antiviral function [19,20]. Irregularities in the extracellular coiled-coil domain are thought to confer flexibility to the molecule [21–23], allowing it to maintain integrity during the intense curvature of the membrane characteristic of virus budding.

Following cell surface tethering, Vpu-defective HIV-1 viral particles are subject to internalization into endosomal compartments, probably resulting in degradation, and dominant-negative mutants of Rab5a can inhibit this process [2,42]. Interestingly, one study identified BCA2/rabring 7, a putative endocytic E3 ubiquitin ligase, as a tetherin-interaction factor capable of enhancing the internalization and degradation of tethered HIV-1 virions from the cell surface [43]. An unresolved question is whether the linkage of tetherin with the actin cytoskeleton helps to recruit tetherin to sites of virus budding or plays a role in re-internalization of tethered virions [17,25].

## 4. Viral Strategies to Counteract Tetherin

The targeting of the host-derived lipid envelope of viruses by tetherin, rather than a specific virally-encoded structure, means that viruses cannot simply mutate their structural proteins to evade it. For an enveloped virus to successfully produce cell-free progeny virus, therefore, it must evolve a counter-strategy, or bud from regions of the cell membrane devoid of tetherin.

There are now several examples of specific countermeasures that have evolved in diverse mammalian viruses to overcome restriction by tetherin [44]. To date, seven mammalian virus-encoded proteins have been reported to counteract tetherin: HIV-1 Vpu, HIV-2 Env, SIV Env, SIV Nef, SIV Vpu, KSHV K5, and the Ebola glycoprotein. All target tetherin differently to achieve the same purpose: the physical separation of tetherin from the site of assembling virions, usually resulting in cell surface downregulation. Tetherin antagonists are often (but not always) species-specific, and in the case of primate lentiviruses this interplay between host antiviral factor and virus-encoded countermeasure may have had profound effects on the zoonotic transmission of these viruses and their adaptation to new hosts.

### 4.1. Human and Simian Immunodeficiency Viruses

**Figure 2:**
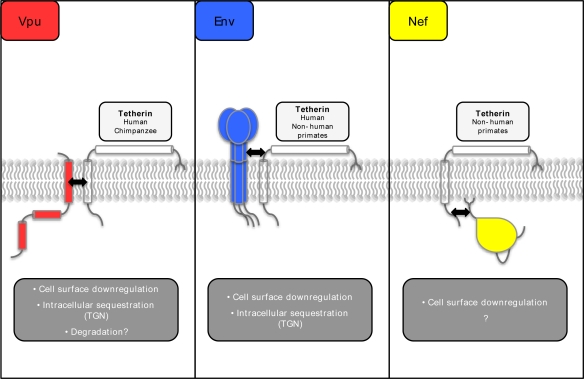
Schematic representation of tetherin and its lentiviral antagonists. The black arrows indicate regions of interaction between tetherin and each lentiviral antagonist, as detailed in the text. The tetherin species specificity of each lentiviral antagonist is indicated in the light grey boxes. Where known, the mechanism(s) by which the antagonists counteract tetherin are detailed in the dark grey boxes.

Tetherin antagonism is a highly conserved attribute amongst primate lentiviruses, implying that evading tetherin is essential for replication of these viruses *in vivo*. Remarkably, the ability to counteract tetherin has arisen in three different primate lentiviral proteins (Vpu, Nef and Env), with the adaptation of these proteins seemingly related to the natural history of cross-species transmission and species-specific differences in primate tetherins (Figure 2). Amongst the six major lineages of primate lentiviruses, only two lineages contain Vpu in their genome, the SIVcpz/HIV-1 lineage and certain members of the SIVsyk lineage that include the SIVgsn sublineage (SIVmus, SIVmon, and SIVgsn), as well as the SIVden isolate.

### 4.2. HIV-1 Vpu

The prototype viral tetherin countermeasure, HIV-1 Vpu, is a small oligomeric transmembrane protein that resides in infected cells predominantly within the TGN and the endosomal system [4,45]. For many years the two known functions of Vpu were the degradation of CD4, and the enhancement of virus release from certain cell types. The former function is well-defined and entails the rapid proteasomal degradation of newly synthesized CD4 molecules to prevent their interference with the trafficking of viral envelope proteins (reviewed in [46]). The nature of the latter function has become clearer since the identification of tetherin as the cellular factor responsible for impeding the release of Vpu-defective HIV-1 mutants. The mechanism by which Vpu antagonizes tetherin is still matter of debate, and to date all experiments studying the molecular basis of this process have been conducted with Vpu proteins derived from Clade B laboratory isolates. It is clear that Vpu expression induces tetherin accumulation in the TGN and that cytoplasmic tail truncations of Vpu that exhibit defects in localisation to the TGN are impaired in antagonism [47]. Moreover, Vpu expression causes a downregulation of cell-surface tetherin levels [3], and the total level of cellular tetherin are decreased in the presence of Vpu in most cellular systems [15,48–51]. However, whether degradation and downregulation of tetherin are absolutely required for Vpu antagonism of tetherin-mediated restriction is less apparent.

#### 4.2.1. Vpu Interaction with Tetherin

Several studies have demonstrated that HIV-1 Vpu and human tetherin physically interact via their respective transmembrane domains [52–57]. This interaction is highly species-specific as single point mutations in the transmembane domain of tetherin render it resistant to Vpu-mediated antagonism [12,15]. Reciprocally, mutations in the transmembrane domain of Vpu diminish its ability to interact with and antagonize tetherin [54]. Interestingly mutations in both proteins’ transmembrane domains that abolish physical interaction lie along single faces of the respective membrane spanning helices [54,56]. This, and the fact that Vpu transmembrane domain mutants that are defective for tetherin interaction are distinct from those that contribute to Vpu’s multimerization or putative ion channel function [54,57], are suggestive that Vpu targets tetherin as a monomer.

#### 4.2.2. The Role of Tetherin Degradation in Antagonism by Vpu

Tetherin is degraded both in cells transfected to express Vpu in *trans* and in cells infected with wild-type HIV-1 [15,48–51]. Tetherin levels in cell lysates can be restored by both proteasomal [15,48,49,58] and endolysosomal inhibitors [50,51,53] leading to debate as to whether tetherin is degraded in lysozomes or by cytoplasmic proteasomes after extraction from intracellular membranes. Because inhibition of tetherin degradation in infected cells by proteasomal inhibitors requires prolonged exposure that is known to deplete cytoplasmic ubiquitin, the more likely situation is that tetherin is degraded by an ubiquitin-dependent endosomal degradation, recently furthered by the implication of HRS and the ESCRT pathway in tetherin turnover [59]. Moreover data supporting proteasomal-dependent degradation of Vpu have in general come from the use of exogenously expressed epitope-tagged tetherin, which often results in a “backing-up” of immature tetherin molecules in the ER and subsequent ER-associated degradation, suggesting that initial reports of the 20S proteasome being directly involved in tetherin degradation are artifactual [60].

The expression of Vpu induces tetherin ubiquitination, with the human tetherin cytoplasmic tail possessing both lysine and serine/threonine residues that can act as ubiquitin acceptors in the presence of Vpu [37,58,61]. The most likely E3-ligase candidate for direct tetherin ubiquitination is the β-transducin repeat-containing protein 2 (β-TrCP) subunit of a Skp1-Cullin1-F-box ubiquitin ligase complex. As observed with Vpu-mediated CD4 downregulation, recruitment of β-TrCP-SCF-Skp 1-Cul1-F-box is required for tetherin degradation. β-TrCP 1 and 2 bind to a highly conserved phosphorylated serine motif (DSGNES) in the Vpu cytoplasmic tail, and are linked to the rest of the E3 ligase complex via an F-box motif [62,63]. Although disruption of the interaction between β-TrCP2 and the cytoplasmic tail of Vpu reduces the capacity of Vpu to enhance virus release [49–51,53,55], there is increasing evidence to show that Vpu’s anti-tetherin mechanism does not necessarily require degradation of the restriction factor. For instance, mutation of both serine residues within the Vpu DSGNES motif preclude binding to β-TrCP and prevent cell surface downregulation of tetherin, yet this mutant Vpu retains some ability to promote virus release [3,51]. Furthermore, while mutation of lysine residues in the cytoplasmic tail of human tetherin renders the protein resistant to Vpu-induced degradation, these proteins are still sensitive to Vpu-mediated antagonism [37,58]. Additionally, one study demonstrated that in infected T-cell lines, tetherin surface expression was only mildly downregulated despite efficient virus release [64].

#### 4.2.3. Intracellular Sequestration as a Mechanism for Antagonism

If tetherin degradation is dispensable for Vpu activity, the most likely mechanism for antagonism of its function is its physical compartmentalization away from budding virions at the cell surface. Many studies have shown that tetherin is downregulated from the cell surface in response to Vpu expression, leading to less incorporation into virions [19,41]. Vpu does not affect the rate of internalization of cell surface tetherin [51,55,60], but rather sequesters *de novo* synthesized or recycling tetherin away from the plasma membrane, probably in the TGN [55,58,65]. This sequestration is sufficient to block the restriction activity of tetherin, and the appropriated tetherin is presumably redirected to the lysosomal compartment for degradation, leading to net depletion of tetherin from the cell surface [60]. This Vpu-mediated sequestration of tetherin may also be ubiquitin dependent. Recently serine- and threonine-linked ubiquitination of tetherin has been implicated in inactivation and surface downregulation by Vpu [61], although whether this is demonstrable at physiological tetherin expression levels is unclear. In some cases, this trapping antagonism mechanism may be augmented by concomitant Vpu-dependent β-TrCP-dependent degradation of tetherin, suggesting that Vpu uses more than one mechanism to counteract tetherin restriction [55,65]. A recent study suggested that HRS, an ESCRT-associated factor that binds ubquitinated cargo, interacts with Vpu/tetherin complexes and was required for antagonism in addition to its clear role in tetherin degradation [59]. However, a potential confounding issue with these observations is that HRS depletion has a marked inhibitory effect on HIV-1 particle production irrespective of whether cells express tetherin or not.

The Vpu proteins from SIVgsn (greater spot-nosed monkey), SIVmus (mustached monkey), SIVmon (Mona monkey) and SIVden (Dent’s Mona monkey) are capable of counteracting tetherin of their simian hosts [66,67]. In contrast, the Vpu from SIVcpz (chimpanzee), the immediate precursor of HIV-1, which shares a common ancestry with SIVgsn/mus/mon Vpu, cannot [66,67]. SIVcpz is in fact a recombinant virus derived from two lineages of SIV and as outlined below, tetherin antagonism has developed separately in two other lentiviral genes. Furthermore, the re-adaptation of HIV-1 Vpu to human tetherin may have had profound consequences for the spread of these viruses in humans.

### 4.3. SIV Nef

Many SIV strains do not encode a Vpu protein. Rather, the Nef protein of SIVmac (macaque), and also of SIVagm (African green monkey), SIVsm (sooty mangagey), SIVblu (blue monkey), and SIV cpz (chimpanzee), can enhance virus release from cells expressing the tetherin proteins derived from their simian hosts [66–69]. Nef is a myristoylated adaptor protein that localizes to the cytosolic face of cellular membranes. This immunomodulatory protein is known to remove cell-surface proteins involved in immune recognition, including CD4, MHC class I and II [70]. Many SIV Nef proteins also induce the cell-surface downregulation of primate tetherin molecules and this downregulation correlates with enhanced viral particle release [69]. However, the underlying mechanistic details of this process remain to be determined.

None of the SIV Nef proteins are able to counteract human tetherin, and this specificity maps to five amino acids (G/DDIWK) in the cytoplasmic domain of simian tetherin that are missing from the human orthologue [16,67–69], leading to speculation that prehistoric infection with viruses encoding Nef-like tetherin antagonists may have selected this deletion. The reinsertion of this motif into human tetherin is sufficient to render it sensitive to SIVmac Nef [68]. Likewise, Nef proteins from both HIV-1 and HIV-2 are unable to antagonize human tetherin, but retain some activity against the rhesus protein [68,69].

### 4.4. HIV-2 and SIV envelope proteins

The third primate lentiviral protein in which tetherin antagonism has been described is the envelope glycoprotein encoded by HIV-2. As with Vpu, the ability of HIV-2 Env to promote virus release from certain cell types was recognized long before this was attributed to the antagonism of tetherin [71]. HIV-2 Env is able to directly interact with tetherin, and like Vpu promotes its cell surface downregulation and sequestration in intracellular compartments, thus excluding tetherin from the site of virus assembly and budding [31,65,72,73]. The exact determinants of tetherin antagonism in HIV-2 Env have yet to be deciphered, but it is clear that a highly conserved endocytic sorting motif (GYxxϕ) in the gp41 cytoplasmic tail is essential. This motif binds to the clathrin adaptor complex AP-2, and presumably facilitates the re-direction of tetherin molecules away from the plasma membrane, sequestering them in a perinuclear compartment [31,65,72,73]. Several studies have implicated the ectodomain of gp41 in tetherin antagonism [31,72,74], but which regions are important for direct interactions and which are needed to maintain a specific structural conformation of the Env complex is unclear. In this respect, proteolytic processing of the envelope into the subunits gp120 and gp41 is also required, as the unprocessed form (gp160) is unable to promote virus release [31,75]. Mutations in the ectodomain of human tetherin render HIV-2 Env incapable of counteracting restriction [76] (specifically, an alanine to aspartic acid substitution at position 100 [14]), supporting the model of an interaction between the ectodomains of both proteins.

Interestingly, there are two documented cases of SIV Env proteins able to antagonise tetherin. The first is an Env protein from a virus originally isolated from a naturally infected tantalus monkey [76]. This SIVtan Env shows a similar ability to HIV-2 Env and HIV-1 Vpu to exclude tetherin from the site of viral budding, resulting in a reduction in surface tetherin levels [14]. The laboratory strain of SIVtan was first isolated from the tantalus monkey by co-culturing monkey and human lymphocytes, and the Nef protein from this virus does have weak activity against its host species tetherin [16]. This raises the possibility that this human cell type adaptation has influenced its ability to antagonise human tetherin, and may not be reflective of Env-mediated antagonism in the natural host. The second case of an SIV Env tetherin antagonist involves a pathogenic *nef*-deleted SIVmac virus isolated from experimentally infected rhesus macaques. Nef-defective mutants of SIVmac are generally attenuated, maintaining a low level chronic infection that does not usually progress to AIDS. However, on occasion pathogenic revertants have been isolated. Upon investigation of the determinants of re-acquired pathogenicity, it was shown that the Env protein had acquired the ability to overcome restriction of virus release specifically by an allele of rhesus tetherin [77,78]. The exact determinants of tetherin antagonism in the SIV Env protein were mapped, and it is of note that they are different from those implicated in the HIV-2 Env [78]. While both Envs require the membrane-proximal GYxxϕ endosomal sorting motif in the gp41 cytoplasmic tail, the remainder of the cytoplasmic tail is dispensable in HIV-2 Env [31,72,79], with the ectodomain postulated to interact with tetherin [31,72,74]; for the *nef*-deleted SIVmac, the ectodomain of Env is dispensable and activity maps to several amino acid substitutions in the C-terminal cytoplasmic tail, and grafting of the tail onto a heterologous membrane protein (CD4) is sufficient to generate a chimeric tetherin antagonist [78]. Unlike HIV-2, SIVmac Env-mediated tetherin interaction and targeting is highly species-specific and is determined by residues flanking the tetherin di-tyrosine endocytic sorting motif, which is also essential for antagonism. There is no effect of the A100D determinant in the tetherin coiled-coil. Thus, the acquisition of function in the SIV Env protein represents an independent and distinct parallel of the evolution of the tetherin antagonistic property of HIV-2 Env.

### 4.5. Tetherin and HIV-1 and HIV-2 Zoonoses and Pathogenesis

Of the four HIV-1 groups (M, N, O and P) that arose from independent zoonoses of SIVcpz, only Vpu proteins of HIV-1 group M and a few strains from group N appear able to efficiently counteract tetherin [66]. Group M has given rise to the HIV-1 pandemic, whilst group O has caused localized epidemics predominantly in Cameroon where it represents 1–2% of the total HIV-1 incidence. Very few isolates of Groups N and P have been sequenced. The relative inefficiency of HIV-1 Group O transmission despite maintaining the ability to cause AIDS, has led to speculation that adaptation of Vpu to human tetherin was an essential step in the establishment of the HIV-1 human pandemic. In the few sequences available for HIV-1 group N, by contrast, tetherin antagonism is variable, but CD4 degradation (present in SIVcpz Vpu) was lost, suggesting that the pressure to adapt to human tetherin in Group N may have disabled this function. Analysis of the TM domains of M, N and O and SIVcpz Vpu proteins reveals that the SIVcpz and Group O TMs differ significantly along their length including in positions known to be important of Group M Vpu function [66]. For SIVcpz Vpu, adaptation of the TM domain is sufficient to confer targeting of human tetherin [16]. Finally, while Group O Vpu proteins lack tetherin antagonism, it is not known whether the virus has, like HIV-2, acquired this function in its envelope protein. There is one report of an HIV-1 group M virus (AD8) with a Vpu-like activity associated with its envelope [80], but to date this observation has not been confirmed in the light of the discovery of tetherin.

It is clear that the Env protein of HIVs and SIVs has the capacity to evolve anti-tetherin activity. This may represent a “reserve” measure in certain situations, as demonstrated by the fact that the HIV-2 Env and an SIVmac Env have gained this function independently and in different regions of the protein. Neither virus was able to enlist Nef to overcome tetherin in its host species: clearly in the *nef*-deleted SIVmac this is because the virus was engineered to lack Nef; for HIV-2 this was a consequence of the human tetherin lacking the five amino acids that confer susceptibility to Nef. Likewise, both viruses are descendents of an SIV lineage that does not encode a Vpu protein. Therefore, Env acts as a third means of counteracting tetherin and establishing infection in a new host. We can only speculate as to whether this has a cost for the virus. In the case of HIV-1 and SIV, tetherin counter-strategies are adopted by accessory proteins that play multiple roles in the modulation of the host environment *in vivo*. In contrast, for HIV-2/SIV Envs, this role is undertaken by a major structural protein, responsible for the entry of the virus into target cells whilst under pressure to constantly evolve to evade attack by the humoral immune response. Furthermore, as Env appears to counteract tetherin by chaperoning and sequestering it in intracellular compartments, this might decrease the levels of infectious virus produced due to a reduced availability of Env protein during assembly. Thus, it is possible that maintaining tetherin antagonistic ability in the Env protein has consequences for viral fitness. Whether this contributes to the lower virulence of HIV-2 is unknown.

### 4.6. Filoviruses: Ebola GP

Ebola virus encodes 7 genes, of which the viral glycoprotein (GP), found on the surface of virions and responsible for mediating target cell entry, is able to antagonize tetherin [33]. Through a mechanism that is currently unclear, Ebola virus GP appears to counteract tetherin without removing it from the cell surface [81]. Furthermore, the Ebola GP does not seem to require specific tetherin sequences for its activity, as it is able to counteract an artificial tetherin, and to date is the only viral tetherin antagonist able to do so [81]. Supporting a less specific mode of action, Ebola GP is also able to antagonize mouse tetherin, a protein possessing only 36% sequence homology to primate tetherins [33]. Thus, Ebola GP seems to have a broader activity than the lentiviral antagonists, perhaps reflecting the breadth of mammalian host species infected by the Ebola virus.

### 4.7. Herpes Viruses: K5 KSHV

Before its identification as an antiviral factor, tetherin had been identified in a proteomic screen for novel targets of K5, a membrane-bound RING-CH (MARCH) domain E3 ubiquitin ligase encoded by the Kaposi's sarcoma-associated herpesvirus (KSHV; also known as human herpesvirus 8 (HHV8)) [82]. K5 is an immuno-modulator that mediates the downregulation of a variety of cell-surface molecules involved in the immune recognition of virally infected cells, such as MHC class I proteins, adhesion molecules and NK receptor ligands [83]. K5 exerts its effects by directly ubiquitinating the cytoplasmic tails of its target proteins to induce ESCRT-dependent degradation. K5 induces a species-specific downregulation of human tetherin from the cell surface followed by its lysosomal degradation, and in its absence the release of progeny KSHV virions from tetherin positive cells is inhibited [36,37]. This K5-mediated tetherin degradation is ESCRT-dependent and requires the direct ubiquitination of a lysine residue at position 18 in the cytoplasmic tail of tetherin [36,37], and unlike Vpu this ubiquitin-coupled degradation is essential for K5 to counteract tetherin function. MARCH ligase homologues are found in several γ2 herpesviruses and poxviruses although whether tetherin antagonism is a conserved function is unknown. Herpesviruses have a complex envelopment strategy involving budding and fusion of immature viruses through the nuclear membrane, followed by ESCRT-dependent budding through internal membranes. The implication of tetherin in restriction of KSHV particle release suggests that all herpesviruses may be susceptible to its antiviral activity. Given the proportion of the genomes of these viruses accounted for by immune evasion genes, it is likely that many other herpesvirus-encoded proteins with anti-tetherin functions await discovery.

## 5. Tetherin and Cell-to-Cell Transmission

HIV-1 and other human retroviruses spread within a host both through cell-free virus dissemination and through direct cell-to-cell contact via the virological synapse (VS) [84,85]. Formation of the virological synapse between infected and target cells is an active process, analogous to the formation of the immunological synapse, involving polarized cytoskeletal remodeling and enrichment of viral proteins and cellular receptors at the site of cell contact. Viral production in the infected cell is polarized towards the site of contact with the target cell, and several recent studies have confirmed the presence of tetherin at the VS [86–88]. Early studies of Vpu-defective HIV-1 replication in T cell lines suggested that while virus production was inhibited, viral spread in the culture was not [4,89–92]. Moreover, selection for HIV-1 variants that spread more efficiently by cell-to-cell spread results in inactivating mutations in Vpu [93]. Therefore re-evaluation of VS-transfer has become of interest recently with respect to the influence of tetherin on this process. However, so far opposing results have been described.

Tetherin-mediated retention of mature virions on the cell surface might inhibit cell-to-cell virus transfer due to aggregation and reduced infectivity of the virions [87,94] or could promote cell-to-cell transfer by providing a concentration of immobilized, infectious virions at the focal point of virus production [86]. Indeed, as the recent literature supports both scenarios it is likely that the outcome is influenced by cell type, modulation of tetherin expression levels by interferon, and the kinetics of the process itself. For example, it has been observed that in macrophages the replication of a virus expressing a Vpu severely impaired in its ability to counteract tetherin was significantly compromised compared to the wildtype virus, whereas replication of the same Vpu-defective virus was not significantly diminished in CD4+ T cells [95]. Furthermore, despite apparently contrasting results with regards to the effect of tetherin on cell-to-cell virus transmission, all studies demonstrated that the reductions in cell-free virus production caused by tetherin were far more effective than any reduction seen in cell-to-cell spread [86,87,94]. The possibility emerges, therefore, that cell-to-cell spread may allow the systemic spread of virions that would otherwise be thwarted by tetherin, particularly considering that no retrovirus beyond the lentivirus genus has been described to encode a tetherin antagonist. Since tetherin antagonism is highly conserved this would imply that, for primate lentiviruses at least, efficient replication and transfer *in vivo* must balance cell-free and cell-to-cell spread, particularly under conditions of high interferon induction (such as acute HIV-1 infection).

Another intriguing possibility is whether tetherin plays a role in the structure of the VS itself. While Vpu mediates tetherin downregulation in infected T cells, in one study this effect appeared delayed [86], and tetherin knock-down also interfered with wild-type virus transfer. Whether this is related to the situation in polarised epithelial cells, wherein tetherin has been shown to interact with the underlying actin cytoskeleton via the BAR-RacGAP protein RICH2 and is concentrated at the apical surface of the cells, remains to be seen [25].

## 6. Tetherin and the Wider Antiviral Immune Response

All studies on tetherin thus far have addressed its effects on virus replication and virion production *in vitro*. However the importance of tetherin in the global immune response to viral infection has yet to be addressed. There are several points at which the actions of tetherin may influence other components of the immune response, all of which may account for the pressure to develop tetherin antagonists over and above its inhibitory activity on virion production.

### 6.1. Antigen Presentation

Viral particles targeted to degradative compartments by tetherin may have consequences in antigen presenting cells, such as macrophages and dendritic cells. For example, in macrophages, the localization of HIV-1 assembly is complex. Virions assemble and accumulate in deep invaginations of the plasma membrane, where they might be sequestered and released on contact with T cells across synaptic structures [96,97]. In addition, Vpu-defective HIV-1 virus particles accumulate in phagosomal structures derived from phagocytic events at the plasma membrane [98]. Thus it is likely that tetherin-HIV interactions result in differential subcellular accumulation of newly assembled virions. This raises the possibility that in antigen presenting cells, such as macrophages, which are targets for HIV-1 infection *in vivo*, tetherin-restricted virions could be targeted for proteolytic destruction in phagosomes and viral components processed for antigen presentation. If so, tetherin-mediated enhanced presentation of viral components to the adaptive immune system will be important in the generation and augmentation of an adaptive immune response.

### 6.2. Enhanced Humoral Recognition of Infected Cells

A further, more general effect of tetherin-restriction of particle release, is the “visibility” that this may give an infected cell to adaptive humoral responses. Depending on the length of exposure to the extracellular milieu, the cell surface-tethered virions themselves may be targets for enhanced antibody deposition. Therefore, virions trapped on the cell surface (and the cell itself) may be subject to complement-mediated destruction or direct cytotoxicity by phagocytes and natural killer cells and the enhanced inflammatory signals that these activities stimulate.

### 6.3. Tetherin as a Ligand for Other Immune Receptors

Recent data have suggested a role for tetherin as a regulator of the interferon response to tumor cells. Specifically, tetherin can act as a ligand for the leukocyte inhibitory receptor ILT7, both of which are expressed on plasmacytoid dendritic cells (pDCs) [11]. This interaction in *cis* on pDC surfaces inhibits signaling through Toll-like receptors 7 and 9 (TLR 7/9) to their respective ligands. Detection of viral nucleic acid by TLRs 7 and 9 induces an interferon response in pDCs, and these cells are recognised as being among the most potent producers of type I IFN during viral infection [99]. While the production of IFN by pDCs induces the upregulation of tetherin on cells in the vicinity, the engagement ILT7 by tetherin inhibits the production of IFN and inflammatory cytokines by pDCs in a negative feedback loop [11]. However ILT7 expression is restricted to pDCs. Does tetherin affect TLR function differently in the absence of ILT7 or in *trans*? Lysosomal degradation of viral particles releases virion components that are ligands for endosomal TLRs. Tetherin restriction of viral particles may therefore lead to increased recognition of viral nucleic acid by TLRs 7 and 9 either in *cis* or *trans*, and in turn upregulate the host response to viral infection by increasing the expression of IFN, inflammatory cytokines and molecules involved in viral immune surveillance. Furthermore tetherin may itself possess an intrinsic signaling capacity. In a large-scale screening study it was identified as a potent inducer of NF-kB [100]. It will be interesting to see, therefore, what the inflammatory consequences of this may be.

## 7. Concluding Remarks

In the last few years, much understanding of tetherin structure and function has been gleaned, particularly with respect to its interactions with HIVs and SIVs, and has led to suggestions that it has acted as a powerful selective pressure on primate lentiviruses. However, the seeming simplicity of its mechanism underlines its potential to act as a potent generalized inhibitor of enveloped virus release. Furthermore, virus/tetherin interactions *in vivo* are likely to lead to further immune activation that may augment and modulate adaptive antiviral responses. The high degree of positive selection on tetherin during mammalian evolution, and the coding capacity that several diverse enveloped viruses have given to develop proteins that specifically target it, imply that tetherin is an important antiviral weapon in the arsenal of the innate immune response.
